# Digital Phenotyping of Geriatric Depression Using a Community-Based Digital Mental Health Monitoring Platform for Socially Vulnerable Older Adults and Their Community Caregivers: 6-Week Living Lab Single-Arm Pilot Study

**DOI:** 10.2196/55842

**Published:** 2024-06-17

**Authors:** Sunmi Song, YoungBin Seo, SeoYeon Hwang, Hae-Young Kim, Junesun Kim

**Affiliations:** 1 Department of Health and Environmental Science Undergraduate School Korea University Seoul Republic of Korea; 2 Department of Physical Therapy College of Health Science Korea University Seoul Republic of Korea; 3 Department of Public Health Sciences Graduate School Korea University Seoul Republic of Korea; 4 Department of Healthcare Sciences Graduate School Korea University Seoul Republic of Korea; 5 BK21FOUR: L-HOPE Program for Community-Based Total Learning Health Systems College of Health Science Korea University Seoul Republic of Korea; 6 Department of Health Policy and Management College of Health Science Korea University Seoul Republic of Korea

**Keywords:** depression, monitoring system, IoT, AI, wearable device, digital mental health phenotyping, living lab, senior care, Internet of Things, artificial intelligence

## Abstract

**Background:**

Despite the increasing need for digital services to support geriatric mental health, the development and implementation of digital mental health care systems for older adults have been hindered by a lack of studies involving socially vulnerable older adult users and their caregivers in natural living environments.

**Objective:**

This study aims to determine whether digital sensing data on heart rate variability, sleep quality, and physical activity can predict same-day or next-day depressive symptoms among socially vulnerable older adults in their everyday living environments. In addition, this study tested the feasibility of a digital mental health monitoring platform designed to inform older adult users and their community caregivers about day-to-day changes in the health status of older adults.

**Methods:**

A single-arm, nonrandomized living lab pilot study was conducted with socially vulnerable older adults (n=25), their community caregivers (n=16), and a managerial social worker over a 6-week period during and after the COVID-19 pandemic. Depressive symptoms were assessed daily using the 9-item Patient Health Questionnaire via scripted verbal conversations with a mobile chatbot. Digital biomarkers for depression, including heart rate variability, sleep, and physical activity, were measured using a wearable sensor (Fitbit Sense) that was worn continuously, except during charging times. Daily individualized feedback, using traffic signal signs, on the health status of older adult users regarding stress, sleep, physical activity, and health emergency status was displayed on a mobile app for the users and on a web application for their community caregivers. Multilevel modeling was used to examine whether the digital biomarkers predicted same-day or next-day depressive symptoms. Study staff conducted pre- and postsurveys in person at the homes of older adult users to monitor changes in depressive symptoms, sleep quality, and system usability.

**Results:**

Among the 31 older adult participants, 25 provided data for the living lab and 24 provided data for the pre-post test analysis. The multilevel modeling results showed that increases in daily sleep fragmentation (*P*=.003) and sleep efficiency (*P*=.001) compared with one’s average were associated with an increased risk of daily depressive symptoms in older adults. The pre-post test results indicated improvements in depressive symptoms (*P*=.048) and sleep quality (*P*=.02), but not in the system usability (*P*=.18).

**Conclusions:**

The findings suggest that wearable sensors assessing sleep quality may be utilized to predict daily fluctuations in depressive symptoms among socially vulnerable older adults. The results also imply that receiving individualized health feedback and sharing it with community caregivers may help improve the mental health of older adults. However, additional in-person training may be necessary to enhance usability.

**Trial Registration:**

ClinicalTrials.gov NCT06270121; https://clinicaltrials.gov/study/NCT06270121

## Introduction

Over the past 2 decades, there has been a notable increase in geriatric depression and other psychiatric disorders, coinciding with a global rise in life expectancy and population aging [1-4]. Since 2009, South Korea has had the highest suicide rate for older adults among the Organisation for Economic Co-operation and Development (OECD) countries. This is attributed to elevated levels of geriatric depression, economic poverty, and social isolation resulting from the rapid nuclearization of the family [5]. With the rapid aging of the global population, caring for older family members with mental disorders has become an overwhelming task for younger generations [6]. Despite the increasing role of community services, there are significant budget shortages in local communities and government health departments, as well as a lack of skilled geriatric labor to meet the needs of these older adults with mental disorders.

The digitalization of mental health screening and intervention is expected to provide innovative solutions to the challenges in mental health care for older adults. For example, digital phenotyping can facilitate the early detection of depression and help reduce the high rate of undiagnosed depression (50%) among older adults [7]. Digital phenotyping of mental health is defined as the “moment-to-moment quantification of the individual-level human phenotype of mental health status in real-life contexts using data collected from personal digital devices” [8]. Recent studies have suggested the potential of digital phenotyping for depressive symptoms using ecological momentary assessments, including self-reports of depressive mood [9]. Beyond momentary self-reports, digital sensing technologies enable unobtrusive passive sensing of depressive symptoms through smartphone apps, wearable sensors, and the Internet of Things. The Internet of Things, a ubiquitous network of interconnected devices, facilitates seamless data collection and intelligent monitoring and management to ensure users’ health and safety [10-13]. However, previous studies have primarily examined digital phenotypes of depressive symptoms in young adults or small groups of patients with depression. This has created challenges in applying digital phenotyping technologies to older adults [3].

The underrepresentation of older adults in digital mental health care research is due to several barriers hindering their participation in studies involving novel digital technologies. First, older adults often have sensory and cognitive impairments that necessitate the use of different design principles than those effective for young or middle-aged adults [14-16]. For example, older adults generally prefer displays with simple layouts and multimodal command functions, such as voice commands in addition to touch screens. Second, implementing digital health care services for older adults should involve both family and community caregivers, who often do not live with the older adult, as well as support from multiple community health care institutions [17,18]. When learning to use new technology, older adults require repeated in-person assistance and educational materials tailored to low digital literacy levels [14,15]. Digital health care services are most likely to benefit older adults when they can connect them to the necessary health care services within the community. Third, the living environments of older adults often hinder their use of digital mental health care services, as a significant proportion (40%-60%) may not have an internet connection or access to personal computers and other mobile devices [19,20].

These functional, social, and environmental barriers underscore the necessity for proof-of-concept and feasibility trials for geriatric mental health care services among older adults and community caregivers in their natural living environments, utilizing living labs [3,18]. Living labs are defined as “user-centered, open innovation ecosystems based on a systematic user co-creation approach, integrating research and innovation processes in real-life communities and settings to create sustainable impact” [21]. Living labs are essential for designing solutions tailored to the needs of older adults. Digital mental health services will not be acceptable or sustainable unless they are designed to be compatible with the cognitive and physical capacities of older adults, as well as their natural living environments, and the working environments of community caregivers. This compatibility can be ensured through living lab testing [22,23].

This study aims to address the gap in current geriatric health literature by testing the feasibility of a digital mental health monitoring platform. This platform could be integrated with existing community senior care services, aiming to prevent and detect early signs of mental health decline in socially isolated older adults. We conducted a single-arm, nonrandomized living lab pilot study involving 25 socially vulnerable older adults. These individuals received personalized daily health monitoring in their natural living environments, both during and after the COVID-19 pandemic. Additionally, the monitoring results of the older adult participants were shared with their community caregivers (n=16) and a managerial social worker at a community senior welfare center (hereafter referred to as the “community center”). This information was utilized by caregivers during their regular in-person senior caregiving services and emergency responses. The study assessed pre- and postintervention changes in mental health indicators (eg, depressive symptoms and sleep quality) and the usability of the monitoring platform among older adults. This evaluation aimed to test both the health-enhancing effects and the usability of the platform. Additionally, beyond feasibility testing, the research investigated whether utilizing digital biomarkers of geriatric depression detected by a wearable sensor could predict daily fluctuations in depressive symptoms among older adults.

## Methods

### Overview

This single-arm, nonrandomized living lab pilot study was undertaken as part of a broader research endeavor aimed at developing a sustainable digital health monitoring platform. The platform is designed to be integrated with a community-based public senior care service, with the goal of enhancing the mental and physical well-being of older adults, both during and following the COVID-19 pandemic. Previously, we conducted formative research using surveys with older adults (n=99) and focused group interviews involving older adults (n=16), community caregivers (n=12), and social workers (n=3) at the community center. The objective was to identify the primary health concerns among older adults, understand the workflow of community caregiving services, and determine the necessary structures and features for the health monitoring service platform. The present digital health monitoring platform incorporates a smartphone chatbot, a smartwatch (Fitbit Sense; Google Inc.), and a motion-sensing camera (Azure Kinect SDK 1.3.0; Microsoft Corporation) installed in the home environment. This platform is designed to enhance the self-care capabilities of older adults by delivering daily updates on their mental and physical health status compared with their baseline averages established during the initial week of the living lab. Additionally, the platform aims to bolster the social support network of older adults by sharing their daily health status information and health emergency alerts with their community caregivers. These caregivers offer regular in-person senior caregiving services to older adult users through the community center. Furthermore, the information was shared with the managerial social worker at the community center to provide assistance in case of any health emergencies during and after the COVID-19 pandemic. Before the pilot study, we conducted an informal prepilot test involving study staff (n=6) to ensure the overall functionality of mobile apps and processing algorithms. The results from the motion-sensing camera, which was integrated with experimental sessions outside the living lab, are reported separately from this study [24]. Trained research assistants, along with a community caregiver, visited older adult participants in their homes to obtain informed consent and install a mobile app on the users’ smartphones and digital devices. Participants were requested to complete surveys at home both before and after the living lab activities, aimed at gauging the usability of the platform and assessing their mental and physical health status. The primary randomized controlled trials featuring both intervention and comparison groups have been registered (registered with Clinicaltrials.gov; registration number NCT06270121); however, this pilot study did not include a control group.

**Figure 1:**
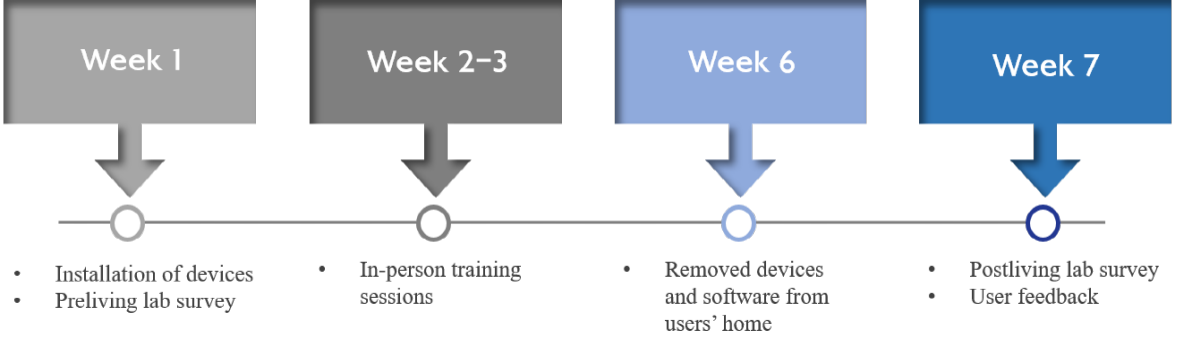
Timeline of the 6-week pilot living lab procedure.

Figure 1 illustrates the timeline of the living lab study procedure, conducted from September 2022 to August 2023. The living lab spanned 6 weeks, commencing with a 1-week adaptation period. Older adults were instructed to launch the smartphone app in the morning, which would prompt them to engage with a chatbot inquiring about their well-being and presenting 2 questions regarding daily depressive symptoms. Following interaction with the chatbot, participants could access their daily health status information, including stress levels (measured via the high frequency [HF] measure of heart rate variability [HRV]), sleep quality (indicated by total sleep time and sleep fragmentation), and physical activity (PA) levels (tracked by step count) from the previous day. This information was compared with their own average during the initial week of the living lab. Voice recordings, daily health status updates, and real-time emergency alerts of the older adult participants were transmitted to their corresponding community caregivers and the managerial social worker through the web or app interface. Throughout the duration of the living lab, participants were instructed to wear a smartwatch continuously, except during battery charging periods.

Trained research assistants conducted surveys at the homes of older adults, both before and after the living lab, to assess significant changes in pilot trial outcomes, such as depressive symptoms, sleep quality, and system usability. During the posttest survey, participants were also queried about the frequency and types of functions they utilized on the platform during the living lab period, and any additional feedback to enhance the platform for the main trials.

To facilitate participants’ acclimatization to the digital monitoring app and devices, and to mitigate the risk of missing data, a member of the research staff visited participants in their homes for 2-3 additional in-person training sessions. These sessions focused on guiding participants on how to use the verbal surveys (Textbox 1) and digital devices effectively, as well as how to check their daily health status. These training sessions were conducted during the second and third weeks of the living lab. If a participant’s data were missed for 3 consecutive days, study staff promptly contacted the participant via phone to emphasize the significance of responding to verbal surveys or wearing the smartwatch. Additionally, they offered assistance in resolving any technical issues hindering the older adult’s participation. Furthermore, community caregivers played a vital role in assisting older adults in adapting to the platform’s usage.

**The methods for assessing daily depressive symptoms via the mobile app chatbot.**

**The 9-Item Patient Health Questionnaire on depressive symptoms**

- Do you feel down, depressed, or hopeless today?
- Do you feel little interest or pleasure in doing things today?
- Have you had trouble falling or staying asleep, or sleeping too much today?
- Are you feeling tired or having little energy today?
- Have you experienced poor appetite or overeating today?
- Do you experience trouble concentrating on things today?
- Are you feeling bad about yourself or that you are a failure or have let yourself or your family down?
- Are you moving or speaking so slowly that other people could have noticed?

**5 items on daily greetings (recommended by community caregivers during the formative research)**

- Did you sleep well last night?
- Have you eaten your meal?
- How are you feeling? Are you feeling pain in any part of your body?
- What are you planning to do today?
- Do you need to go to the hospital today?

**An example daily voice survey scenario combining 1 randomly selected greeting item and 2 randomly selected items from the 9-Item Patient Health Questionnaire**

Good morning, Ma’am!

[Greeting item] Did you sleep well last night? (Recording)

[PHQ-9 #1] Do you feel down, depressed, or hopeless today? (Recording)

[PHQ-9 #2] Are you moving or speaking so slowly that other people could have noticed? (Recording)

Thank you! Have a good day.

### Ethics Approval

The study protocol received approval from the Institutional Review Board of Korea University (approval number KUIRB-2021-0324-02).

### Recruitment

Adults older than 65 years, along with their community caregivers, were recruited from a community center in Seoul, South Korea. A meeting was convened with the community caregivers and managers at the community center to elucidate the study’s objectives and procedures, solicit participation as community caregivers, and seek assistance in recruiting older adult participants. Based on the formative research findings, community caregivers can be described as predominantly middle-aged women (mean age 58.04, SD 3.17 years), with a gender composition of women only. On average, they possessed 1-2 years of work experience. Each caregiver was responsible for providing in-person caregiving services to multiple older adults, with a maximum caseload of 16 individuals. Caregivers who expressed interest in participating in the study with their older adult service recipients explained the study protocol to the older adult during their subsequent regular in-person visits. If both the older adult and their community caregiver were interested in participating, the study staff arranged a home visit to obtain informed consent from the older adult, accompanied by their caregiver.

The inclusion criteria encompassed older adults receiving in-person senior care services due to socioeconomic vulnerability, particularly those living alone with low income. Additionally, participants were required to use a Samsung Galaxy smartphone, as the mobile app was exclusively developed for Android smartphones, which are widely utilized by older adults in South Korea. Exclusion criteria encompassed cognitive and functional impairments that could impede study participation (such as hearing loss), as well as individuals residing with others in the same household. This exclusion was due to technical challenges associated with motion-sensing camera detection, which were pertinent to the broader study. Community caregivers offering in-person public senior care services at the community center were deemed eligible to participate in the study. A managerial social worker responsible for overseeing the in-person senior care service at the community center was also recruited to evaluate the website of the monitoring platform.

Of the 39 older adults initially recruited for the study, 8 declined participation, primarily citing busyness and concerns about the long-term use of the platform as reasons. Consequently, the study proceeded with the consented participation of the remaining 31 older adults.

### Patient Health Questionnaire-9

The 9-item Patient Health Questionnaire-9 (PHQ-9) was used to evaluate daily depressive symptoms through the smartphone app chatbot, reflecting insights from the formative research of the larger study. This decision was informed by older adult users’ challenges with touch screen usage and their preference for verbal communication features over visual displays. The PHQ-9 is a widely recognized tool utilized to identify mild and clinical depressive symptoms in nonpsychiatric settings [25,26]. One item from the PHQ-9 questionnaire, specifically concerning suicidal thoughts (“Have you had thoughts that you would be better off dead, or of hurting yourself?”), was omitted due to the potential risk of eliciting negative thoughts, particularly among individuals who may be at risk for depression.

**Figure 2:**
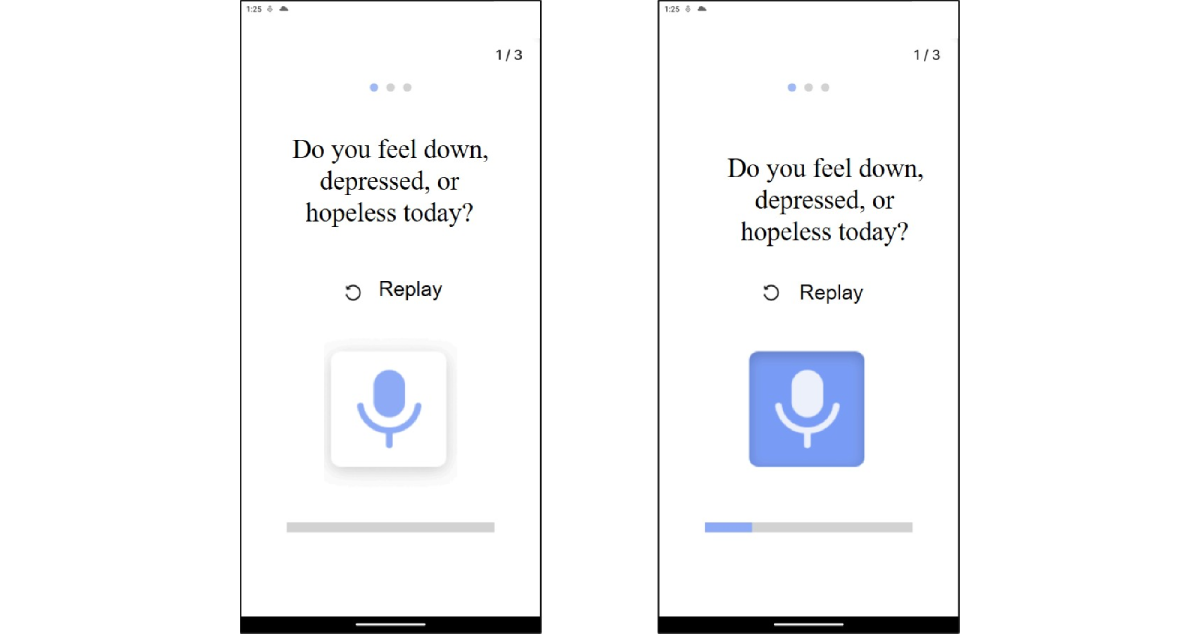
Mobile app function for collecting daily depressive symptoms; the chatbot voice asked 2 randomly selected PHQ-9 items and older adult participants’ answers were automatically recorded. The blue bar and the blue microphone icon indicate that voice recording has been activated. PHQ-9: 9-item Patient Health Questionnaire.

In addition to the PHQ-9 items, we created a chatbot script for daily greetings and safety checks, incorporating 5 commonly used questions recommended by community caregivers. Older adult participants were instructed to open the mobile app every morning, triggering an automated conversation with the chatbot (upon the first daily app launch). Voice recording was activated for 30 seconds after each question, and the recorded file was instantly uploaded to the website accessible to community caregivers (Figure 2). These voice recording files were monitored during the living lab and coded later to identify the presence of daily depressive symptoms (1=depressive symptoms indicated on at least one PHQ-9 item, 0=no reports of depressive symptoms) by 2 research assistants (YBS and SYH) independently. Any discrepancies were resolved through discussions among the 2 coders and an experienced supervisor.

### Smartwatch-Derived Measures

#### Daily Sleep Quality

Sleep quality was evaluated using several metrics, including total sleep time, sleep fragmentation index, and the number of long fragmentation episodes. To validate our sleep calculation algorithm, we examined baseline data on participants’ usual sleep time and wake time using items from the Pittsburg Sleep Quality Index (PSQI). Our algorithm commenced at 6 PM and concluded at noon the following day to encompass all sleep periods. Additional adjustments were implemented for 1 older adult whose day and night cycles had reversed, allowing for the capture of daytime sleep periods (n=1). The Fitbit algorithm categorized the activity level for each minute the older adult wore the sensor as either asleep or awake. Furthermore, the classification data were prescreened using the following 7 criteria to improve the accuracy of actigraphy-assessed sleep detection based on previous studies [27,28]: (1) if the previous 4 minutes were categorized as awake, the initial minute of the sleep period was adjusted to sleep; (2) if the previous 10 minutes were identified as awake, the subsequent 3 minutes were adjusted to awake; if more than (3) 15 minutes before and (4) after a sleep period lasting less than 6 minutes were classified as awake, then the period of less than 6 minutes of sleep was adjusted to awake; (5) if more than 20 minutes before and after a sleep period lasting less than 10 minutes were categorized as awake, the period of less than 10 minutes of sleep was adjusted to awake. The total time in bed was computed as the duration between the initiation and cessation of the sleep cycle; (6) the sleep onset time was determined as the first time block featuring at least 10 minutes of uninterrupted sleep; and (7) the sleep offset time was determined as the final 10 minutes of uninterrupted sleep before rising from bed. The total sleep time was computed by aggregating the minutes spent asleep from the sleep onset to the sleep offset. The sleep fragmentation index was determined by dividing the number of times the participant awakened for more than 1 minute by the total sleep time [27]. Sleep efficiency was calculated by dividing the total sleep time by the duration of time spent in bed [27].

#### Heart Rate Variability

HRV was evaluated using both time and frequency domain indicators. These indicators were computed every 5 minutes, around the clock, using the Python-based (Python Foundation) open-source program code Aura-healthcare [29]. The code converted heart rate data to R-R intervals, which represent the time elapsed between 2 successive R-waves in the QRS signal on the electrocardiogram. These intervals are known to be influenced by the activity of the sinus node and autonomic nerve stimulation [30]. Next, the code was used to compute the time domain indicators, which included the SD of the N-N intervals, the normalized or filtered R-R intervals, and the root mean square of successive differences. Additionally, frequency domain indicators were derived, such as the ratio of low frequency (LF) to HF, and HF after applying fast Fourier transformation. This transformation categorized the power of heart rate into HF, LF, and very-low-frequency components. The characteristics and reliability of HRV assessments using Fitbit have been documented in a previous study involving a diverse population [31].

#### Physical Activity Indices

PA levels were evaluated using Fitbit’s indicators for steps taken and minutes spent engaging in light, moderate, and intense activity each day throughout the living lab participation. Fitbit uses a 3-axis accelerometer to track steps and PA by analyzing the frequency, duration, intensity, and patterns of movements. Previous studies have confirmed the accuracy of Fitbit activity data [32-34].

### Provision of Individualized Daily Health Status Feedback Via the Mobile App

**Figure 3:**
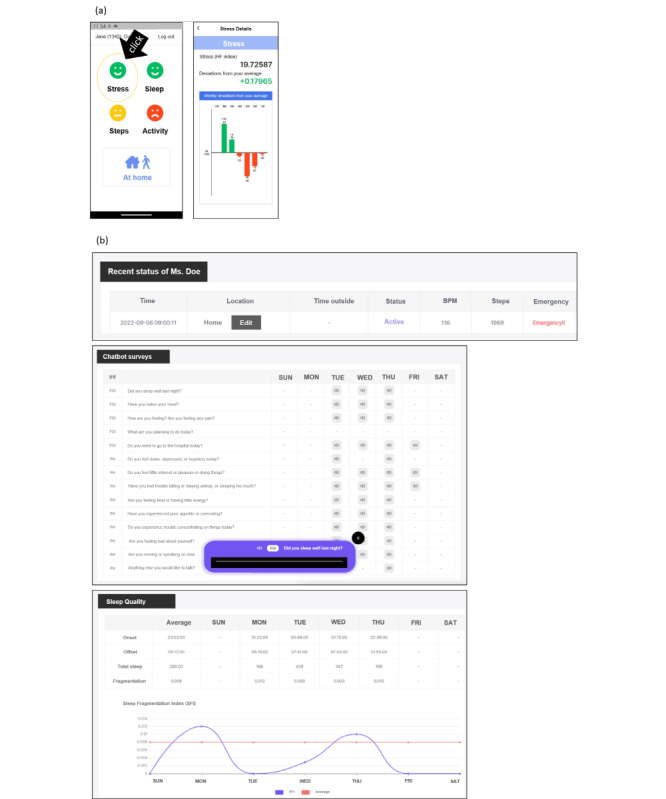
Example of customized daily health status feedback on stress, sleep, steps, and activity using traffic signal colors and detailed information via the mobile app for (A) the older adult users and (B) the web application for the community caregivers and the managerial social worker.

The mobile app designed for older adults and their community caregivers delivered personalized daily health status feedback on stress, sleep, steps, and activity. This feedback was presented using traffic signal colors and accompanied by detailed information, as depicted in Figure 3A. Using the traffic signal colors, a green face within the health status for stress, steps, and activity domain indicated a value higher than 1 SD of the user’s average in that domain. A yellow face denoted a value within +1 SD or –1 SD of the average of the health status domain score. A red face indicated a value lower than 1 SD of the average level. The ranges for the green and red faces were inverted for sleep (as measured by sleep fragmentation) compared with the other measurable categories, as a higher value indicates more fragmented sleep. Clicking each face on the initial page (Figure 3A on the left side) advanced the user to the next page, where details (Figure 3A on the right side) were provided regarding the differences between today’s value in the domain compared with the average during the first week. Additionally, a weekly graph was included to illustrate the pattern of changes in the weekly values.

The web application designed for the community caregiver and the managerial social worker (Figure 3B) facilitated centralized monitoring by community caregivers, allowing for swift emergency responses to older adults during and after the COVID-19 pandemic. Additionally, it enabled the sharing of daily health status updates. The emergency signal would be triggered if an older adult remained inactive for over 8 hours and/or if their heart rate fell below 30 bpm or rose above 140 bpm.

### Pre- and Postsurvey Measures

#### Geriatric Depression Scale

The 15-item version of the Geriatric Depression Scale (GDS) [35,36] was used to evaluate levels of depressive symptoms in older adults during the pre- and postliving lab surveys. Scores ranged from 0 to 15, with cutoff scores of ≥5 and ≥10 indicating a risk for mild and severe depression, respectively.

#### Pittsburgh Sleep Quality Index

Subjective sleep quality was evaluated using the Pittsburgh Sleep Quality Index (PSQI) in the pre- and postliving lab surveys [37,38]. The PSQI comprises 19 items and assesses 7 components of sleep over the past month. The total PSQI score was utilized to indicate the overall level of subjective sleep quality, with a cutoff score of 5 or more indicating a risk for sleep disorders.

#### System Usability Scale

The *System Usability Scale* (SUS) was used to gauge the older adults’ experience levels and perceived difficulty in using the digital monitoring platform during the pre- and postliving lab surveys [39]. The scale includes questions regarding the participant’s frequency of digital technology usage and their perception of its ease of use, among others. Responses are rated on a 5-point scale ranging from 0=strongly disagree to 4=strongly agree.

Many older adult participants encountered challenges in identifying digital technology to base their answers on for this scale during the preliving lab survey. This survey was conducted on the day when the digital health monitoring platform was installed. To assist participants in answering the survey, examples of digital devices such as smartphones or kiosks at hospitals were provided. Postliving lab assessments of the SUS were conducted based on the participants’ experiences with the developed health monitoring platform.

In addition to the SUS, participants were queried about the number of days per week they utilized the platform and the number of functions they accessed on the platform during the postliving lab survey. Furthermore, at the posttest, participants were requested to offer qualitative feedback aimed at enhancing the platform for future trials. This feedback was transcribed verbatim by a research assistant.

#### Covariates

Covariates were selected a priori, drawing from existing literature that outlines demographic and health risk factors associated with daily depressive symptoms among older adults [7,40]. Alongside baseline depression levels, demographic and health factors known to be correlated with depression (eg, age, sex, and chronic health conditions) were utilized as covariates.

### Statistical Analysis

After analyzing missing data and assessing the distributions of key variables, descriptive analyses were performed. Group comparisons between depressed and nondepressed older adults were conducted using *t* tests for continuous variables (eg, age, BMI, PSQI total score, daily depressive symptoms, wearable sensor assessments of HRV, sleep, and PA indicators), chi-square tests for categorical variables (eg, sex, education, income, chronic disease, sleep disorder categories), and Fisher exact tests for smoking status. Subsequently, bivariate correlations were explored between baseline depression and person mean variables (daily depressive symptoms and wearable sensor assessments) over 5 weeks, excluding the initial week for adaptation.

**Figure 4:**
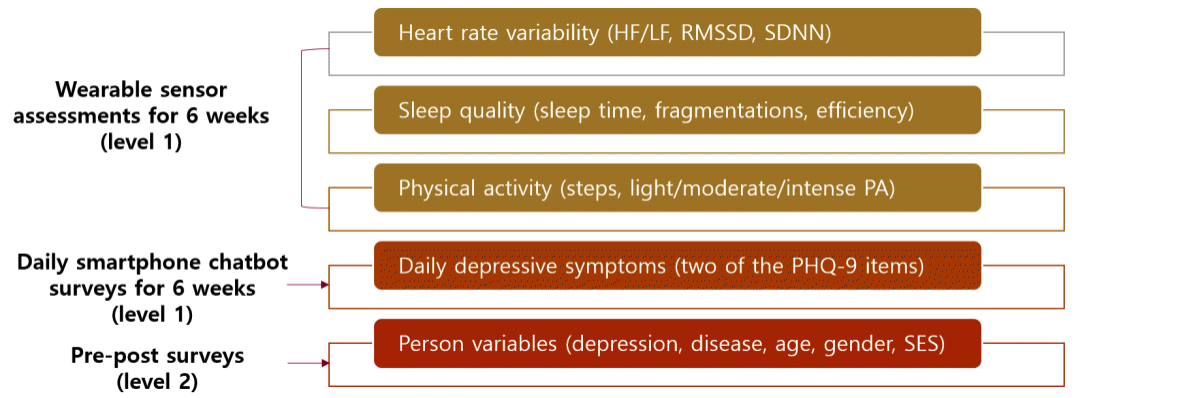
Multilevel data structure of this study with wearable sensor and daily depressive symptom chatbot survey data as level 1 and pre- and posttest survey data as level 2. HF: high frequency; LF: low frequency; PA: physical activity; PHQ-9: 9-item Patient Health Questionnaire; RMSSD: root mean square of successive differences; SDNN: standard deviation of N-N intervals; SES: socioeconomic status.

To identify digital biomarkers for daily depressive symptoms in older adults, we used multilevel modeling (MLM) analysis. This method allowed us to scrutinize the daily shifts in depressive symptoms, as well as the smartwatch-detected metrics of sleep quality, HRV, and activity levels over time. We utilized SAS PROC MIXED (SAS Institute) for this analysis. Applying MLM analysis to daily depression and smartwatch data facilitates the identification of antecedents and correlates of daily depressive symptoms. It also allows for the use of participants as their own controls [41]. In the multilevel models, the level 1 equation investigated whether daily sleep, HRV, and PA indices predicted the likelihood of reporting any depressive symptoms on the same day and the following day. To assess the primary impact of smartwatch indices on daily depressive symptoms, the main effect models for each of the sleep/HRV/activity model indices were utilized as level 1 predictors, with daily depressive symptoms on the same day or the next day serving as level 1 outcome variables. In all MLM analyses, the level 2 equations incorporated personal characteristics, including participants’ demographic and health covariates. Daily measures were person mean centered, while level 2 covariates were grand mean centered. This approach allows for the interpretation of estimates as the probability of daily depressive symptoms when there are deviations in the predicting digital biomarker variable from the participant’s own average across the 5-week living lab period. The multilevel data structure of this study is illustrated in Figure 4. A type I error rate of 0.05 was established for statistical analyses, except for the MLM analyses, where Bonferroni and Holm corrections for multiple testing were applied, resulting in a type I error rate of 0.005.

The pre- and posttest results were assessed using a paired *t* test for variables demonstrating normal distributions, such as system usability. However, because of their skewed distributions, depressive symptoms and sleep quality were analyzed using the Wilcoxon signed rank test. To delve deeper into the age moderation effects on the pre- and posttest results for usability, repeated-measure ANOVA was applied to examine potential differences in system usability between the oldest group (>75 years old) and the rest (65-74 years old).

### Power Analysis

Power analysis for multilevel model design is recognized for its complexity. Previous studies have revealed a compensatory relationship between the number of participants and the number of observations per participant [42,43]. We gathered 807 days of observational data from 25 older adults, averaging 32.28 days per participant (range 8-40 days). The variability in the number of available days stemmed primarily from 2 reasons: participants’ nonadherence to data collection protocols (eg, forgetting to wear the Fitbit) and the scheduling preferences for installing and removing digital devices from their homes (eg, 1 participant needed to schedule device removal 1 week later than others because of personal reasons). A recent simulation study proposed that a sample size of 25 combined with continuous data collection over 30 days yields acceptable performance levels in terms of parameter estimations [42].

### Data Exclusion and Missing Data

Out of the initial 31 adults who participated, data from 6 participants were excluded from the study. They dropped out after the installation of sensing devices due to experiencing inconvenience and difficulties in using mobile apps and digital devices. Among the initial group, 25 older adults provided their responses to daily verbal surveys and wearable sensor data. These data were included in the multilevel analyses, focusing on the primary outcome of daily depressive symptoms. Of 808 days of assessments (with a mean of 32.32 days per participant), there were 186 days (23% of total days) with missing daily verbal survey data, primarily as a result of participants forgetting to respond or no responses being recorded. Additionally, there were 269 days (33.3%) with missing HRV data, 440 days (54.5%) with missing sleep measures, and 298 days (36.9%) with missing steps data. These missing data were primarily attributed to participants forgetting to wear a smartwatch during the day or night, or experiencing issues with charging the device. Previous studies on missing data analysis suggest that multilevel analyses using full information maximum likelihood estimation methods are generally robust against estimation biases arising from data with partial missingness [44]. Regarding pre-post test effects, 1 participant did not complete the posttest survey as a result of long-term travel for family matters, resulting in data from 24 older adults being included in the analyses.

## Results

### Descriptive Overview

The demographic and health characteristics of the older adult living lab participants are detailed in Table 1. The participants exhibited characteristics typical of older adults, with a mean age of 76.40 (SD 4.23) years. Additionally, they displayed social vulnerabilities concerning education and income levels. Among the participants, there were more women (n=19) than men (n=6). On average, participants reported having 4 chronic disease conditions, with 3 (12%) of the 25 participants reporting depressive disorders and 10 (40%) reporting arthritis or diabetes. The average level of sleep quality was poor, with 22 (88%) participants at high risk for sleep disorders.

Compared with individuals with a low risk of depression at baseline, those with a high risk were more likely to report daily depressive symptoms via verbal surveys. Additionally, they tended to sleep longer but with lower efficiency and were less likely to engage in light, moderate, and intense PA (*P* values ranged from <.001 to.02).

**Figure 5:**
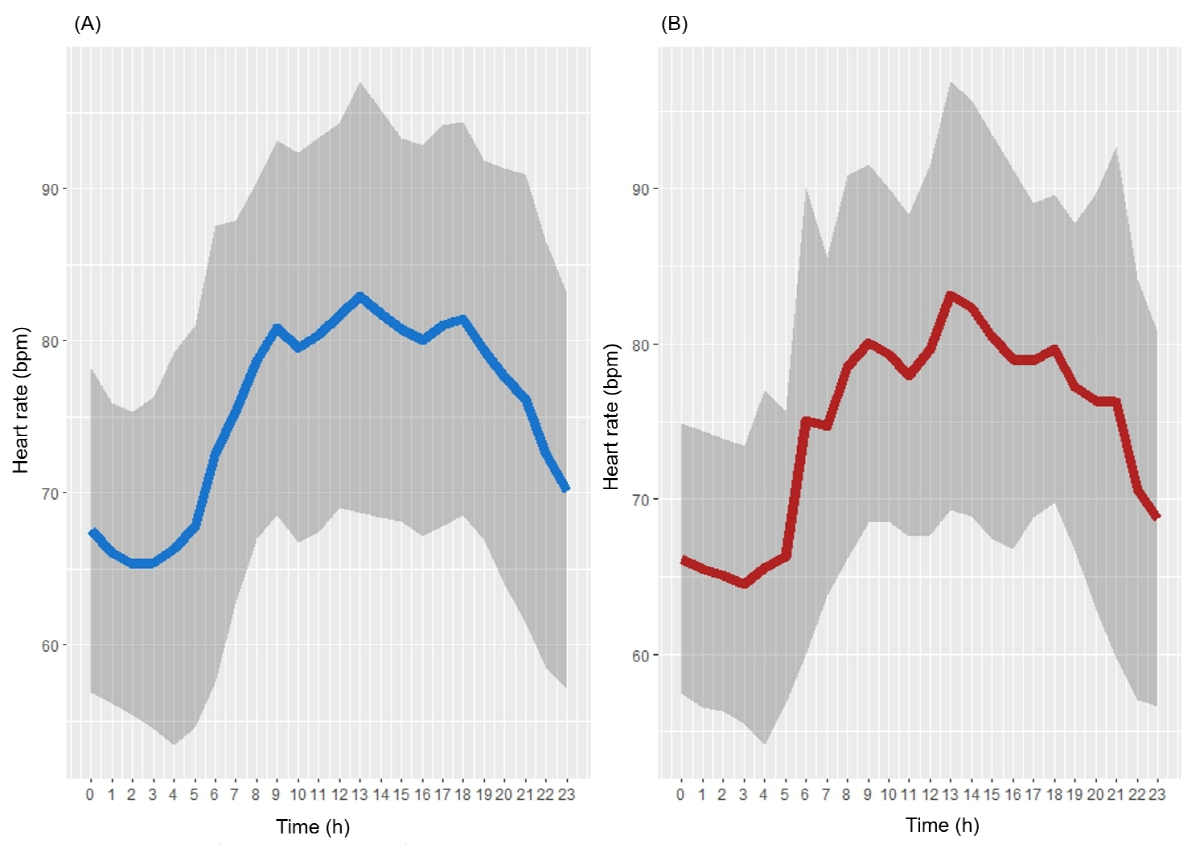
The average 24-hour profiles of heart rate on (A) total days and (B) days with depressive symptoms.

Figure 5 illustrates the 24-hour profile of heart rate on both total days and days when older adults reported depressive symptoms. The patterns of heart rate on days with depressive symptoms seemed to exhibit more variability throughout the day compared with total days.

**Table 1:** Characteristics of older adult living lab participants.

Characteristics				Total (n=25)		Baseline depressive symptoms^a^				*P* value
						No (n=15)		Yes (n=10)		
**Demographic characteristics**										
	Age (years), mean (SD)			76.40 (4.23)		76.40 (4.42)		76.40 (4.17)		>.99
	Sex: women, n (%)			19 (76)		11 (73)		8 (80)		.70
	**Education, n (%)**									.51
		Elementary school or less	14 (56)		7 (47)		7 (70)			
		Middle school	2 (8)		2 (13)		0 (0)			
		High school	5 (20)		3 (20)		2 (20)			
		College or more	4 (16)		3 (20)		1 (10)			
	**Monthly income (Korean won^b^), n (%)**									.95
		Less than 500,000	4 (16)		2 (13)		2 (20)			
		500,001-1,000,000	16 (64)		10 (67)		6 (60)			
		1,000,001-1,500,000	2 (8)		1 (7)		1 (10)			
		1,500,001 or more	3 (12)		2 (13)		1 (10)			
**Health characteristics**										
	**Smoking, n (%)**									.18
		Current smoking	1 (4)		0 (0)		1 (10)			
		Past smoking	6 (24)		6 (40)		0 (0)			
		Never	18 (72)		9 (60)		9 (90)			
	BMI (kg/m^2^), mean (SD)			24.56 (3.94)		24.65 (2.44)		24.43 (5.68)		.89
	**Number of chronic diseases, mean (SD)**			4.20 (1.71)		4.20 (1.37)		4.20 (2.20)		>.99
		Arthritis, n (%)	10 (40)		5 (33)		5 (50)		.49	
		Cardiovascular disease, n (%)	18 (72)		12 (80)		6 (60)		.27	
		Diabetes, n (%)	10 (40)		7 (47)		3 (30)		.40	
		Depression, n (%)	3 (12)		2 (13)		1 (10)		.80	
		Dementia, n (%)	1 (4)		0 (0)		1 (10)		.21	
**Baseline study variables**										
	PSQI^c^ global sleep quality (0-21 points^d^), median (IQR)			8.00 (6.00-10.00)		7.00 (6.00-9.00)		9.00 (6.75-11.25)		.22
	Sleep disorders, n (%) of PSQI>5			22 (88)		12 (80)		10 (100)		.13
	**Digital assessments, person mean (SD)**									
		Daily PHQ symptoms, 0-1	0.37 (0.48)		0.31 (0.46)		0.48 (0.50)		<.001	
	**Heart rate variability, mean (SD)**									
		Low frequency/high frequency	5.94 (1.33)		5.89 (1.30)		6.00 (1.36)		.34	
		High frequency	26.02 (37.14)		23.75 (20.57)		28.71 (49.99)		.14	
		SD of the N-N intervals	103.01 (41.89)		108.81 (44.73)		96.17 (37.22)		<.001	
		Root mean square of successive differences	9.32 (3.87)		9.13 (3.58)		9.55 (4.19)		.21	
	**Sleep, mean (SD)**									
		Total sleep time	319.63 (163.08)		297.98 (166.73)		345.12 (155.30)		.004	
		Sleep fragmentation index	0.02 (0.02)		0.02 (0.02)		0.02 (0.02)		.61	
		Sleep efficiency	65.98 (20.86)		68.31 (21.18)		63.24 (20.20)		.02	
	**Physical activity, mean (SD)**									
		Steps	3207.11 (3901.80)		3512.54 (4024.61)		2839.53 (3724.16)		.05	
		Light physical activity	130.46 (101.55)		148.81 (107.43)		104.19 (86.19)		<.001	
		Moderate physical activity	14.05 (19.34)		15.75 (20.09)		11.62 (17.98)		.02	
		Intense physical activity	21.89 (27.85)		24.40 (26.02)		18.30 (29.98)		.02	

^a^The presence or absence of depressive symptoms was determined based on a score of 5 or higher on the Geriatric Depression Scale.

^b^US $1=1344 Korean won.

^c^PSQI: Pittsburg Sleep Quality Index.

^d^Higher scores indicate worse sleep quality.

**Figure 6:**
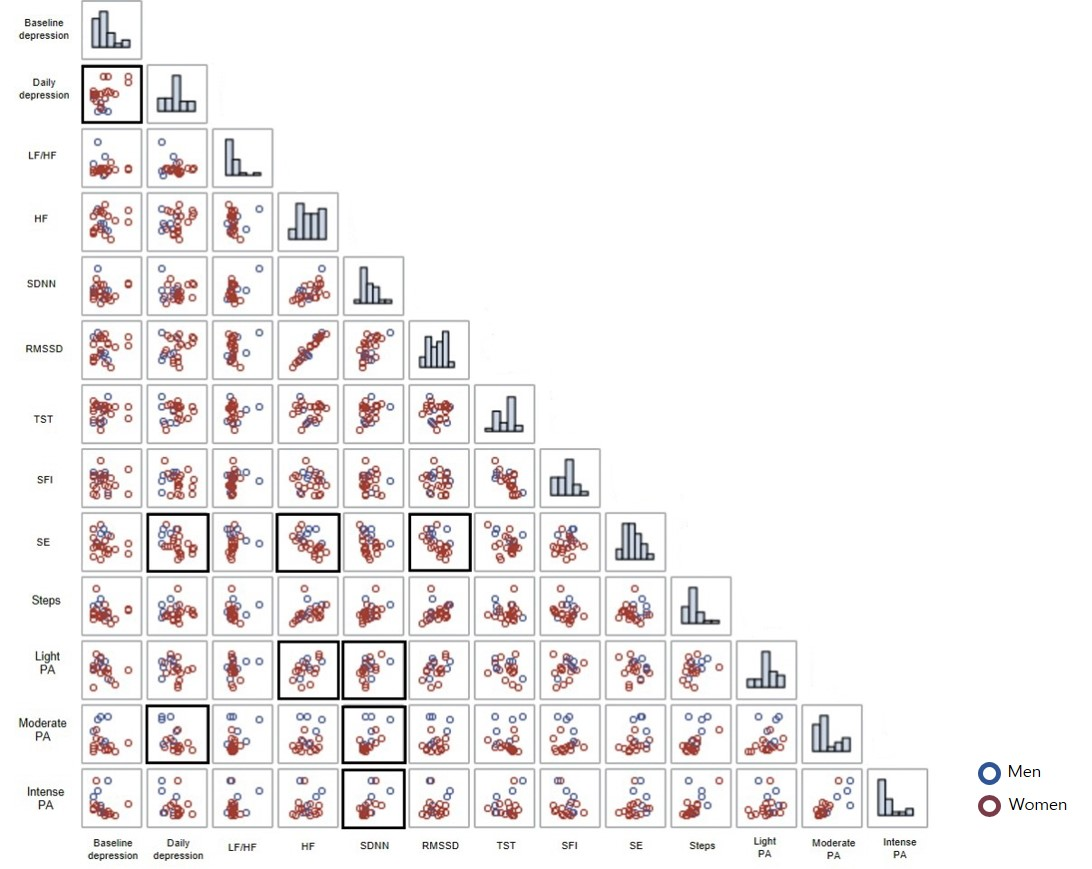
Bidirectional correlations among baseline depression; person mean of daily depression; and digital markers of heart rate variability indicators, sleep indicators, and physical activity measures (bold box indicates significance at *P*<.05; red dots represent women, blue dots represent men. HF: high frequency; LF/HF: low frequency-to-high frequency power ratio; PA: physical activity; RMSSD: root mean square of successive differences; SDNN: standard deviation of N-N intervals; SE: sleep efficiency; SFI: sleep fragmentation index; TST: total sleep time.

Figure 6 displays the results of bivariate correlation analysis among baseline depression measures; the person mean score of daily depressive symptoms; and the smartwatch measures of HRV, sleep quality, and PA. Additionally, distributions of the variables are presented on the diagonals. As anticipated, baseline depressive symptoms were significantly correlated with the person mean variable of daily depressive symptoms (*r*=0.43, *P*=.03). Interestingly, the person mean of daily depressive symptoms exhibited a negative association with daily sleep efficiency (*r*=–0.47, *P*=.02) and the amount of moderate PA (*r*=–0.50, *P*=.02). Furthermore, HRV, sleep, and PA indicators exhibited correlations with each other. Specifically, person mean variables of daily HF and root mean square of successive differences were associated with daily sleep efficiency (*r*=0.58, *P*=.003 and *r*=–0.51, *P*=.009, respectively). Additionally, the person mean levels of daily HRV indicators were associated with the person mean levels of daily PA. HF exhibited a positive correlation with light PA (*r*=0.49, *P*=.03), whereas SD of the N-N intervals was positively associated with light (*r*=0.45, *P*=.05), moderate (*r*=0.54, *P*=.01), and intense PA (*r*=0.59, *P*=.006).

### Primary Outcomes

As depicted in Table 2, the MLM results indicated that both the daily sleep fragmentation index and sleep efficiency significantly predicted the occurrence of daily depressive symptoms measured on the subsequent day, even after adjusting for baseline depression, age, sex, and chronic disease conditions (odds ratio 2.066, 95% CI 1.252-3.411; *P*=.003 for daily sleep fragmentation and odds ratio 0.972, 95% CI 0.955-0.989; *P*=.001 for daily sleep efficiency). In essence, on days following more fragmented sleep or lower sleep efficiency compared with their own average, older adult participants were more inclined to report depressive symptoms via the chatbot survey. Notably, the effects of daily sleep fragmentation and efficiency on daily depressive symptoms remained significant even after applying Bonferroni-Holm corrections for multiple testing. However, none of the daily assessments in HRV, sleep quality, or PA predicted the occurrence of depressive symptoms the following day.

**Table 2:** The concurrent and lagged effect models of daily digital indicators on daily depressive symptoms^a,b,c^.

Digital predictors		Same-day depressive symptoms		Next-day depressive symptoms	
		Odds ratio (95% CI)	*P* value	Odds ratio (95% CI)	*P* value
**Heart rate variability**					
	High frequency/low frequency	0.975 (0.790-1.204)	.82	0.929 (0.751-1.148)	.49
	High frequency	0.987 (0.617-1.579)	.96	0.978 (0.632-1.513)	.92
	SD of the N-N intervals	0.998 (0.990-1.005)	.57	1.001 (0.994-1.008)	.84
	Root mean square of successive differences	0.954 (0.866-1.051)	.34	0.998 (0.924-1.078)	.96
**Sleep**					
	Total sleep time	0.999 (0.996-1.001)	.16	1.001 (0.999-1.003)	.35
	Sleep fragmentation index	2.066 (1.252-3.411)	.003^c^	1.149 (0.750-1.759)	.77
	Sleep efficiency (%)	0.972 (0.955-0.989)	.001^c^	0.999 (0.983-1.016)	.93
**Physical activity**					
	**Steps**	1.000 (1.000-1.000)	.28	1.000 (1.000-1.000)	.21
	Light physical activity	0.999(0.996-1.002)	.48	1.002 (0.998-1.005)	.33
	Moderate physical activity	0.992 (0.971-1.014)	.48	0.985 (0.964-1.006)	.16
	Intense physical activity	0.998 (0.985-1.011)	.73	1.002 (0.989-1.015)	.75

^a^All analyses were adjusted for age, sex, chronic disease conditions, and baseline depression.

^b^All digital predictors were person mean centered so that daily values represent deviations from the individuals’ own means across the participation days.

^c^The Bonferroni-Holm correction suggests that *P* values indicating significance should be <.005, with 11 multiple tests for each of the 2 outcome variables.

### Pre- and Posttest Results

As presented in Table 3, the pre- and posttest results showed significant decreases in depressive symptoms (pre-post difference –1.000, 95% CI –2.000 to 0.000, *W*=143.500, *z*=–1.976, *P*=.048) and improvements in sleep quality (pre-post difference –1.500, 95% CI –3.000 to 0.000, *W*=165.000, *z*=–2.252, *P*=.02) after the 6-week living lab than before. However, there were no significant changes in the levels of usability (pre-post difference –6.354, 95% CI –15.969 to 3.261, t_23_=–1.376, *P*=.18). Age moderation was also not significant in the pre- and posttest changes in usability (*F*_1,22_=437.682, *P*=.20). At the posttest survey, participants reported using the monitoring app a mean of 6.68 (SD 1.44) days per week and utilizing 1.58 functions (SD 1.91) out of a possible 5. These functions included recording verbal surveys for daily depressive symptoms and checking individualized health feedback for stress, steps, physical functions, and sleep time.

In the posttest survey, older adults expressed satisfaction with the chatbot survey, particularly appreciating its inquiries about their daily lives and health conditions. They felt cared for and safe, knowing that their voice message would be delivered to their community caregiver daily. However, older adults also voiced frustrations with smartwatch malfunctions. They experienced technical issues during the living lab period as a result of difficulties in regularly charging the smartwatch, accidentally turning off the smartphone’s Bluetooth connection, and overloading of the central server.

**Table 3:** Changes in depressive symptoms, sleep quality, and usability levels from pre- to posttests.

Variables	Pretest	Posttest	*P* value
Depressive symptoms (Geriatric Depression Scale), median (IQR)^a^	3.000 (1.000-6.000)	1.500 (0.250-5.000)	.048
Sleep quality (Pittsburg Sleep Quality Index), median (IQR)^a^	8.000 (6.000-10.000)	6.000 (4.000-7.750)	.02
Usability (System Usability Scale), mean (SD)^b^	53.333 (24.524)	59.688 (19.620)	.18

^a^Wilcoxon signed rank test was used to analyze depressive symptoms and sleep quality due to their skewed distributions.

^b^Paired *t* test was used to evaluate usability based on a normal distribution of the usability scores.

## Discussion

### Principal Findings

This study explored the viability of digitally phenotyping depressive symptoms and implementing continuous digital mental health monitoring among a small cohort of socially vulnerable older adults (n=25) in their everyday living settings. By conducting daily verbal assessments of depressive symptoms via a chatbot and monitoring daily fluctuations in digital biomarkers using a smartwatch, this study indicates that 2 digital measures of daily sleep quality, namely, sleep fragmentation and sleep efficiency, predicted the occurrence of daily depressive symptoms on the subsequent day. These associations remained significant even after adjusting for potential confounders such as baseline depression, age, gender, and chronic disease conditions. To the best of our knowledge, this study represents the first attempt to explore the digital phenotyping of depressive symptoms in socially vulnerable older adults within their own living environments over an extended duration. Furthermore, the pre- and posttest findings revealed that while depressive symptoms and sleep issues improved following the 6-week platform utilization alongside community caregiver support, no significant enhancement was observed in system usability. Although the current findings are preliminary and lack a comparison group, they suggest the potential health advantages of integrating a digital health monitoring platform for older adults in conjunction with in-person community senior care services.

### Comparison With Prior Work

Through the development of analytic algorithms for continuous sensing of sleep time, fragmentation, and efficiency, this study unveiled that daily alterations in sleep fragmentation and efficiency during the night, relative to an individual’s average, were predictive of daily depressive symptoms among older adults on the subsequent day. These findings are consistent with prior studies that have demonstrated a significant correlation between actigraphy-assessed wake time after sleep onset, sleep efficiency, and 1-time survey assessments of depressive symptoms among adults with a history of clinical depression [12,40,45]. A meta-analysis of 38 studies incorporating actigraphy assessments similarly found significant disparities in longer wake time after sleep onset between patients with depression and healthy controls [46]. Our findings contribute to the existing literature by investigating daily variations in depressive symptoms among socially isolated older adults over an extended period. Moreover, this study examines whether within-person fluctuations in sleep quality forecast ongoing changes in depressive symptoms across consecutive days.

The current findings revealed that neither actigraphy-assessed HRV nor PA measures significantly predicted daily depressive symptoms. Prior research has demonstrated varied associations between HRV and depressive symptoms, depending on factors such as the diagnosis or severity of depression and the cardiovascular health of the population under study. Indeed, a meta-analysis of 21 studies highlighted significant distinctions between individuals with depression and healthy controls [47]. Additionally, a previous investigation involving patients with depression documented a negative correlation between HRV (eg, root mean square of successive differences) and cognitive symptoms of depression (eg, rumination) on the same day [48]. However, such relationships between HRV and depressive symptoms were not observed in adults without clinical depression or those with cardiovascular health issues. For instance, depressive symptoms were not significantly correlated with electrocardiogram-measured HRV in healthy adults [49] or adults at risk of coronary artery disease [50]. The majority of older adults (18/25, 72%) in this study had cardiovascular disease conditions, a prevalence higher than the national average in South Korea (40.36%) and the global prevalence (31.0%-70%) for adults over 70 years [51-53]. Future research with a larger sample of older adults is warranted to investigate the associations between depressive symptoms and HRV among older adults with diverse chronic disease conditions.

Regarding the association between depressive symptoms and PA, in contrast to previous findings, daily fluctuations in PA did not predict daily depressive symptoms in this study. Indeed, a meta-analysis comprising 42 studies utilizing actigraphy or pedometer assessments of PA demonstrated significant associations between average PA levels and depressive symptom severity among adults, regardless of clinical depression status [54]. Additionally, intervention programs aimed at increasing PA were associated with lower levels of depressive symptoms compared with control groups in adult populations without clinical depression [55]. There could be several potential explanations for the lack of association between daily PA and daily depressive symptoms observed in our study. It is plausible that long-term patterns of PA, rather than day-to-day fluctuations, may better explain changes in depressive symptoms. Consistent with this hypothesis, the present findings demonstrated a significant negative correlation between the average levels of depressive symptoms and the average levels of moderate PA across individuals. Future research is warranted to explore potential factors that might elucidate the relationship between daily depressive symptoms and PA. This could include investigating cumulative patterns of PA, the impact of specific types of PA (such as group exercise), or external circumstances that may impede physical activities (such as the COVID-19 pandemic).

Finally, the pre- and posttest findings indicated that older adults exhibited enhancements in depressive symptoms and sleep quality following their utilization of the platform alongside their community caregivers. It is important to note that the participating older adults had previously been receiving in-person senior caregiving services, which entailed regular phone calls and weekly in-person visits for safety checks, as well as assistance with hospital visits if necessary. However, the utilization of the present digital monitoring platform may have empowered community caregivers to optimize the timing of service provision based on when older adults were most in need. For example, an older adult user received red lights on sleep and physical activity via the user app when they had poor sleep and skipped exercise due to a flare-up of chronic disease conditions. Their community caregiver received the same daily feedback and voice recordings (from the respective user) about health issues via the caregiver app. Subsequently, the caregiver phoned or visited each participant to check on the negative health changes for that day. The current findings align with recent studies demonstrating the health-improving effects of digital health care services for older adults. These effects were observed when mental health professionals provided in-person services [56,57] or when community health workers with intensive training [58,59] were connected to the digital service. However, this study makes a unique contribution to the literature by presenting preliminary findings suggesting that community caregivers without a health care specialty or intensive training in mental health care can potentially enhance the mental health of socially vulnerable older adults through the utilization of a digital health monitoring platform.

Contrary to expectations, the results of the usability test in this study suggest that older adults experienced difficulties in using the mental health monitoring system, and these challenges did not improve over time. These findings underscore the importance of offering adequate in-person assistance, including group lessons, in-person training sessions, and troubleshooting visits for older adults when introducing a new digital health care service.

### Limitations

Our findings should be interpreted with caution due to several limitations. First, the sample size was limited to 25 older adults, which may not have provided sufficient power to detect small effect sizes of associations between daily depressive symptoms and sensor-based daily indicators, as well as changes in outcome measures pre- to posttest. The current results may not be generalizable to other older adult populations or other countries due to our deliberate selection of older adults with social vulnerabilities, including those living alone with low income. These individuals may experience greater challenges in accessing digital technologies compared with the overall older adult population [60]. We used MLM to investigate the concurrent digital biomarkers of daily depressive symptoms. However, future studies may benefit from exploring alternative analytic approaches such as machine learning to classify older adults into high- and low-risk groups for depression. Additionally, it is important to acknowledge that the strict implementation of COVID-19–related social restriction policies in 2022 may have influenced the findings of this study.

### Conclusions

This study investigated the feasibility of mental and physical health monitoring platforms for socially vulnerable older adults and their community caregivers in their everyday living environments. Additionally, it explored whether passively sensed measurements of HRV, sleep, and PA predicted daily fluctuations of depressive symptoms in older adults. The findings indicate that older adults successfully utilized the monitoring platform throughout the 6-week study to monitor their daily health status. This information was also shared with community caregivers to enhance the existing senior care service. Additionally, the MLM results revealed same-day associations between daily sleep quality indicators (sleep fragmentation index and sleep efficiency from the previous night) and daily depressive symptoms. The pre- and posttest results indicate that older adults exhibited enhancements in depressive symptoms and sleep quality following the utilization of the monitoring platform, which was integrated with their existing community care services. These findings offer preliminary support for the digital phenotyping of geriatric depressive symptoms using sleep measures obtained from a wearable sensor. We also propose a potential service delivery model for developing a hybrid senior care service that integrates online and offline components. This model leverages existing community-based senior care services to facilitate the early detection and prevention of mental health declines in socially vulnerable older adults.

## Abbreviations

GDS: Geriatric Depression Scale
HF: high frequency
HRV: heart rate variability
LF: low frequency
MLM: multilevel modeling
OECD: Organisation for Economic Co-operation and Development
PA: physical activity
PHQ-9: 9-item Patient Health Questionnaire
PSQI: Pittsburg Sleep Quality Index
SUS: System Usability Scale

## References

[1] (2022). The old-age to working-age ratio will nearly quadruple in Korea by 2060. OECD Library.

[2] Kim GE, Jo M, Shin Y (2020). Increased prevalence of depression in South Korea from 2002 to 2013. Sci Rep.

[3] Fortuna KL, Torous J, Depp CA, Jimenez DE, Areán Patricia A, Walker R, Ajilore O, Goldstein CM, Cosco TD, Brooks JM, Vahia IV, Bartels SJ (2019). A future research agenda for digital geriatric mental healthcare. Am J Geriatr Psychiatry.

[4] World Health Organization (WHO) (2023). Mental health of older adults. WHO.

[5] OECD (2022). Old-age suicide rate is very high in Korea but has been declining. OECD Reviews of Pension Systems: Korea.

[6] World Health Organization (WHO) (2023). Aging and health. WHO.

[7] Zenebe Y, Akele B, W/Selassie M, Necho M (2021). Prevalence and determinants of depression among old age: a systematic review and meta-analysis. Ann Gen Psychiatry.

[8] Kamath J, Leon Barriera Roberto, Jain N, Keisari E, Wang B (2022). Digital phenotyping in depression diagnostics: integrating psychiatric and engineering perspectives. World J Psychiatry.

[9] Sano A, Taylor S, McHill AW, Phillips AJ, Barger LK, Klerman E, Picard R (2018). Identifying objective physiological markers and modifiable behaviors for self-reported stress and mental health status using wearable sensors and mobile phones: observational study. J Med Internet Res.

[10] Onnela J, Rauch SL (2016). Harnessing smartphone-based digital phenotyping to enhance behavioral and mental health. Neuropsychopharmacology.

[11] BinDhim NF, Shaman AM, Trevena L, Basyouni MH, Pont LG, Alhawassi TM (2015). Depression screening via a smartphone app: cross-country user characteristics and feasibility. J Am Med Inform Assoc.

[12] Difrancesco S, Lamers F, Riese H, Merikangas KR, Beekman ATF, van Hemert Albert M, Schoevers RA, Penninx BWJH (2019). Sleep, circadian rhythm, and physical activity patterns in depressive and anxiety disorders: a 2-week ambulatory assessment study. Depress Anxiety.

[13] Sorri K, Mustafee N, Seppänen M (2022). Revisiting IoT definitions: a framework towards comprehensive use. Technological Forecasting and Social Change.

[14] Wildenbos GA, Peute L, Jaspers M (2018). Aging barriers influencing mobile health usability for older adults: a literature based framework (MOLD-US). Int J Med Inform.

[15] Xie B, Charness N, Fingerman K, Kaye J, Kim MT, Khurshid A (2020). When going digital becomes a necessity: ensuring older adults' needs for information, services, and social inclusion during COVID-19. J Aging Soc Policy.

[16] Chen K, Chan AHS (2014). Gerontechnology acceptance by elderly Hong Kong Chinese: a senior technology acceptance model (STAM). Ergonomics.

[17] Willems SH, Rao J, Bhambere S, Patel D, Biggins Y, Guite JW (2021). Digital solutions to alleviate the burden on health systems during a public health care crisis: COVID-19 as an opportunity. JMIR Mhealth Uhealth.

[18] Chen C, Ding S, Wang J (2023). Digital health for aging populations. Nat Med.

[19] Hunsaker A, Hargittai E (2018). A review of Internet use among older adults. New Media & Society.

[20] Delello JA, McWhorter RR (2017). Reducing the digital divide: connecting older adults to iPad technology. J Appl Gerontol.

[21] European Network of Living Labs (ENoLL) (2023). What Are Living Labs?. ENoLL.

[22] Verloo H, Lorette A, Rosselet Amoussou J, Gillès de Pélichy Estelle, Matos Queirós Alcina, von Gunten A, Perruchoud E (2021). Using living labs to explore needs and solutions for older adults with dementia: scoping review. JMIR Aging.

[23] Korman M, Weiss PL, Kizony R (2016). Living Labs: overview of ecological approaches for health promotion and rehabilitation. Disabil Rehabil.

[24] Jo S, Song S, Kim J, Song C (2022). Agreement between Azure Kinect and marker-based motion analysis during functional movements: a feasibility study. Sensors (Basel).

[25] Martin A, Rief W, Klaiberg A, Braehler E (2006). Validity of the Brief Patient Health Questionnaire Mood Scale (PHQ-9) in the general population. Gen Hosp Psychiatry.

[26] Manea L, Gilbody S, McMillan D (2015). A diagnostic meta-analysis of the Patient Health Questionnaire-9 (PHQ-9) algorithm scoring method as a screen for depression. Gen Hosp Psychiatry.

[27] Fekedulegn D, Andrew ME, Shi M, Violanti JM, Knox S, Innes KE (2020). Actigraphy-based assessment of sleep parameters. Ann Work Expo Health.

[28] Webster J B, Kripke D F, Messin S, Mullaney D J, Wyborney G (1982). An activity-based sleep monitor system for ambulatory use. Sleep.

[29] hrv-analysis documentation. Github.

[30] Pappano AJ, Wier WG (2018). Cardiovascular Physiology: Cardiovascular Physiology--E-Book.

[31] Natarajan A, Pantelopoulos A, Emir-Farinas H, Natarajan P (2020). Heart rate variability with photoplethysmography in 8 million individuals: a cross-sectional study. Lancet Digital Health.

[32] Feehan LM, Geldman J, Sayre EC, Park C, Ezzat AM, Yoo JY, Hamilton CB, Li LC (2018). Accuracy of Fitbit devices: systematic review and narrative syntheses of quantitative data. JMIR Mhealth Uhealth.

[33] Redenius N, Kim Y, Byun W (2019). Concurrent validity of the Fitbit for assessing sedentary behavior and moderate-to-vigorous physical activity. BMC Med Res Methodol.

[34] Sushames A, Edwards A, Thompson F, McDermott R, Gebel K (2016). Validity and reliability of Fitbit Flex for step count, moderate to vigorous physical activity and activity energy expenditure. PLoS One.

[35] Yesavage JA, Brink TL, Rose TL, Lum O, Huang V, Adey M, Leirer VO (1982). Development and validation of a geriatric depression screening scale: a preliminary report. J Psychiatr Res.

[36] Jung IK, Kwak DI, Shin DK, Lee MS, Lee HS, Kim JY (1997). A reliability and validity study of Geriatric Depression Scale. J Korean Neuropsychiatr Assoc.

[37] Buysse DJ, Reynolds CF, Monk TH, Berman SR, Kupfer DJ (1989). The Pittsburgh Sleep Quality Index: a new instrument for psychiatric practice and research. Psychiatry Res.

[38] Sohn SI, Kim DH, Lee MY, Cho YW (2012). The reliability and validity of the Korean version of the Pittsburgh Sleep Quality Index. Sleep Breath.

[39] Holden RJ (2020). A Simplified System Usability Scale (SUS) for Cognitively Impaired and Older Adults. Proceedings of the International Symposium on Human Factors and Ergonomics in Health Care.

[40] Kim H, Lee S, Lee S, Hong S, Kang H, Kim N (2019). Depression prediction by using ecological momentary assessment, Actiwatch data, and machine learning: observational study on older adults living alone. JMIR Mhealth Uhealth.

[41] Raudenbush SW, Bryk AS (200). Hierarchical Linear Models: Applications and Data Analysis Methods.

[42] Hecht M, Zitzmann S (2020). Sample size recommendations for continuous-time models: compensating shorter time series with larger numbers of persons and vice versa. Structural Equation Modeling: A Multidisciplinary Journal.

[43] Martens MJ, Logan BR (2021). A unified approach to sample size and power determination for testing parameters in generalized linear and time-to-event regression models. Stat Med.

[44] Graham JW (2009). Missing data analysis: making it work in the real world. Annu Rev Psychol.

[45] Robillard R, Naismith SL, Smith KL, Rogers NL, White D, Terpening Z, Ip TKC, Hermens DF, Whitwell B, Scott EM, Hickie IB (2014). Sleep-wake cycle in young and older persons with a lifetime history of mood disorders. PLoS One.

[46] Tazawa Y, Wada M, Mitsukura Y, Takamiya A, Kitazawa M, Yoshimura M, Mimura M, Kishimoto T (2019). Actigraphy for evaluation of mood disorders: a systematic review and meta-analysis. J Affect Disord.

[47] Koch C, Wilhelm M, Salzmann S, Rief W, Euteneuer F (2019). A meta-analysis of heart rate variability in major depression. Psychol Med.

[48] Ottaviani C, Shahabi L, Tarvainen M, Cook I, Abrams M, Shapiro D (2014). Cognitive, behavioral, and autonomic correlates of mind wandering and perseverative cognition in major depression. Front Neurosci.

[49] Schwerdtfeger A, Friedrich-Mai P (2009). Social interaction moderates the relationship between depressive mood and heart rate variability: evidence from an ambulatory monitoring study. Health Psychol.

[50] Bhattacharyya M, Whitehead D, Rakhit R, Steptoe A (2008). Depressed mood, positive affect, and heart rate variability in patients with suspected coronary artery disease. Psychosom Med.

[51] Aïdoud A, Gana W, Poitau F, Debacq C, Leroy V, Nkodo J, Poupin P, Angoulvant D, Fougère B (2023). High Prevalence of Geriatric Conditions Among Older Adults With Cardiovascular Disease. JAHA.

[52] Oh MS, Jeong MH (2020). Sex differences in cardiovascular disease risk factors among Korean adults. Korean J Med.

[53] Arnold AM, Psaty BM, Kuller LH, Burke GL, Manolio TA, Fried LP, Robbins JA, Kronmal RA (2005). Incidence of cardiovascular disease in older Americans: the Cardiovascular Health Study. J Am Geriatr Soc.

[54] Gianfredi V, Blandi L, Cacitti S, Minelli M, Signorelli C, Amerio A, Odone A (2020). Depression and objectively measured physical activity: a systematic review and meta-analysis. Int J Environ Res Public Health.

[55] Rebar AL, Stanton R, Geard D, Short C, Duncan MJ, Vandelanotte C (2015). A meta-meta-analysis of the effect of physical activity on depression and anxiety in non-clinical adult populations. Health Psychol Rev.

[56] Hong S, Lee S, Song K, Kim M, Kim Y, Kim H, Kim H (2023). A nurse-led mHealth intervention to alleviate depressive symptoms in older adults living alone in the community: a quasi-experimental study. Int J Nurs Stud.

[57] Peng R, Guo Y, Zhang C, Li X, Huang J, Chen X, Feng H (2024). Internet-delivered psychological interventions for older adults with depression: a scoping review. Geriatr Nurs.

[58] Richards D, Timulak L, Doherty G, Sharry J, Colla A, Joyce C, Hayes C (2014). Internet-delivered treatment: its potential as a low-intensity community intervention for adults with symptoms of depression: protocol for a randomized controlled trial. BMC Psychiatry.

[59] Scazufca M, Nakamura CA, Seward N, Moreno-Agostino D, van de Ven P, Hollingworth W, Peters TJ, Araya R (2022). A task-shared, collaborative care psychosocial intervention for improving depressive symptomatology among older adults in a socioeconomically deprived area of Brazil (PROACTIVE): a pragmatic, two-arm, parallel-group, cluster-randomised controlled trial. Lancet Healthy Longev.

[60] Seifert A, Cotten SR, Xie B (2021). A double burden of exclusion? Digital and social exclusion of older adults in times of COVID-19. J Gerontol B Psychol Sci Soc Sci.

